# Developing an Automated Assessment of In-session Patient Activation for Psychological Therapy: Codevelopment Approach

**DOI:** 10.2196/38168

**Published:** 2022-11-08

**Authors:** Sam Malins, Grazziela Figueredo, Tahseen Jilani, Yunfei Long, Jacob Andrews, Mat Rawsthorne, Cosmin Manolescu, Jeremie Clos, Fred Higton, David Waldram, Daniel Hunt, Elvira Perez Vallejos, Nima Moghaddam

**Affiliations:** 1 Specialist Services Nottinghamshire Healthcare NHS Foundation Trust Nottingham United Kingdom; 2 School of Computer Science, University of Nottingham Nottingham United Kingdom; 3 School of Computer Science and Electronic Engineering, University of Essex Essex United Kingdom; 4 Mindtech Medtech Co-operative, University of Nottingham Nottingham United Kingdom; 5 Hilltop Digital Lab Ltd Stockport United Kingdom; 6 Institute of Mental Health University of Nottingham Nottingham United Kingdom; 7 School of English, University of Nottingham Nottingham United Kingdom; 8 Nottingham Biomedical Research Centre Mental Health and Technology Theme University of Nottingham Nottingham United Kingdom; 9 School of Psychology, University of Lincoln Lincoln United Kingdom

**Keywords:** responsible artificial intelligence, machine learning, cognitive behavioral therapy, multimorbidity, natural language processing, mental health

## Abstract

**Background:**

Patient activation is defined as a patient’s confidence and perceived ability to manage their own health. Patient activation has been a consistent predictor of long-term health and care costs, particularly for people with multiple long-term health conditions. However, there is currently no means of measuring patient activation from what is said in health care consultations. This may be particularly important for psychological therapy because most current methods for evaluating therapy content cannot be used routinely due to time and cost restraints. Natural language processing (NLP) has been used increasingly to classify and evaluate the contents of psychological therapy. This aims to make the routine, systematic evaluation of psychological therapy contents more accessible in terms of time and cost restraints. However, comparatively little attention has been paid to algorithmic trust and interpretability, with few studies in the field involving end users or stakeholders in algorithm development.

**Objective:**

This study applied a responsible design to use NLP in the development of an artificial intelligence model to automate the ratings assigned by a psychological therapy process measure: the consultation interactions coding scheme (CICS). The CICS assesses the level of patient activation observable from turn-by-turn psychological therapy interactions.

**Methods:**

With consent, 128 sessions of remotely delivered cognitive behavioral therapy from 53 participants experiencing multiple physical and mental health problems were anonymously transcribed and rated by trained human CICS coders. Using participatory methodology, a multidisciplinary team proposed candidate language features that they thought would discriminate between high and low patient activation. The team included service-user researchers, psychological therapists, applied linguists, digital research experts, artificial intelligence ethics researchers, and NLP researchers. Identified language features were extracted from the transcripts alongside demographic features, and machine learning was applied using k-nearest neighbors and bagged trees algorithms to assess whether in-session patient activation and interaction types could be accurately classified.

**Results:**

The k-nearest neighbors classifier obtained 73% accuracy (82% precision and 80% recall) in a test data set. The bagged trees classifier obtained 81% accuracy for test data (87% precision and 75% recall) in differentiating between interactions rated high in patient activation and those rated low or neutral.

**Conclusions:**

Coproduced language features identified through a multidisciplinary collaboration can be used to discriminate among psychological therapy session contents based on patient activation among patients experiencing multiple long-term physical and mental health conditions.

## Introduction

### Background

One psychological therapist can vary significantly from another in how effective they are for their patients [1,2]. Furthermore, individual psychological therapists do not necessarily, on average, improve their effectiveness with time or experience [3]. In addition, the beneficial effects of psychological therapies have not grown in many areas, and in some cases, effectiveness has declined over time [4,5]. Given that time and experience alone do not seem to improve effectiveness, there are currently few evidence-based means of helping psychological therapists improve their efficacy. This situation is unhelpful for patients, with significant differences in effectiveness among the psychological therapists they may see. It is also unhelpful for psychological therapists and psychological therapy services with few scalable, cost-effective means of supporting practitioners to improve their effectiveness. There have been calls for systematic, objective, and routine means of measuring the quality of psychological therapy content [6,7], and the application of artificial intelligence (AI) may offer part of the solution, especially in combination with text classification and other natural language processing (NLP) techniques.

AI is defined as a form of technology that (1) is to some degree able to perceive the environment and real-world complexity; (2) collects and interprets information inputs; (3) can perform decision-making, including the ability to learn and reason; and (4) can achieve predetermined goals [8]. Increasingly, AI has been used to categorize and evaluate the contents of psychological therapy sessions in research. In face-to-face psychological therapy, supervised learning models have achieved reliable automation of psychological therapy competency assessments, with particular advances in motivational interviewing and more recently cognitive behavioral therapy [9,10]. In messaging- and internet-based psychological therapy, a bottom-up, unsupervised learning approach has been used to identify the types of language used where clinical improvement is significantly more likely and, conversely, where it is less likely [11,12].

There are several potential benefits to these approaches. First, automated evaluation of psychological therapy could offer scalable, routine assessment of psychological therapy interactions where human coding can be too time consuming and costly [13,14]. Second, AI offers the potential to improve identification and verification of prognostic markers in psychological therapy contents, with associated trainable skills for therapists, which may either be difficult to identify from human coding or where important markers are hard to discover because research of sufficient scale is impractical with human raters. Overall, this approach could offer psychological therapists ongoing feedback on their practice, as routinely recommended [15]. This would allow continual improvements in effectiveness when coupled with, for example, deliberate practice techniques to enhance therapeutic microskills [16,17].

However, none of the current uses of AI in psychological therapy contents have focused on patients experiencing multiple comorbidities (or multimorbidity). This is significant, given that differences among therapists are more pronounced among patients with more complex problems, and patients experiencing multimorbidity generally have poorer prognoses [18]. In addition, more active participation and engagement during health care consultations can have an especially positive effect on long-term physical health, mental health, and service use among patients experiencing multimorbidity [19]. This is particularly important because the majority of treatment and care for multimorbid conditions is undertaken by the patients themselves [20]. Furthermore, the ability of patients in this group to self-manage their care is highly affected by clinician responsiveness and interaction style [21,22]. This suggests that specific in-session process markers may be suitable for automated identification and classification in a patient group where psychological therapy is at greater risk of failure, and interaction style can have an important impact on engagement and prognosis. Current evidence has also been largely restricted to either face-to-face psychological therapy or messaging-based treatment. Less attention has been paid to the large and growing use of videoconferencing psychological therapy since the onset of the COVID-19 pandemic [23].

The important issues of algorithmic trust and participatory approaches to development have also not been sufficiently addressed in current applications of AI to psychological therapy. In recent years, significant concerns have arisen regarding the increasing pervasiveness of algorithms and the impact of automated decision-making in health care, alongside the poverty of research into applying AI systems in practice [24]. This means that AI systems are being developed without sufficient involvement or consideration of stakeholders affected by AI decisions. Particularly problematic is the lack of transparency surrounding the development of these algorithmic systems and their use [25].

Within the field of mental health, the engagement and involvement of key stakeholders, including service users, have been identified and recommended as part of the process of developing trustworthy AI applications [26,27]. Stakeholder engagement is one of the pillars of responsible research and innovation [28] and is central to this study to increase the trustworthiness and relevance of emerging AI applications in psychological therapy. As well as increasing trust in AI, the involvement of stakeholders (including service users) can help address systematic biases in AI systems that can replicate human prejudices in the decisions made [29,30]. At this stage in the nascent use of AI for analyzing psychological therapy content, it may be important to establish methods for using AI responsibly in this particular context [31].

A recently developed psychological therapy rating tool may provide an opportunity to address some of the current gaps in the evidence around the use of AI for psychological therapy evaluation. The consultation interactions coding scheme (CICS) [32] was developed to rate individual psychological therapy interactions, turn by turn, based on patient activation. Patient activation has become a significant, well-used, and well-researched concept in health care, particularly for people experiencing multimorbidity [33,34]. Patient activation is the degree to which a person feels confident and able to be actively involved in managing their own health [35]. Patient activation is distinct from other related motivation and engagement constructs because it more specifically focuses on how engagement and motivation are expressed in consultation interactions between health care users and health care professionals [36]. The patient activation measure (PAM) is the established means of assessing patient activation in research and clinical practice [37]. However, as a retrospective questionnaire, the PAM may not be able to fully inform interventions designed to increase patient activation, which often involve adjusting interaction style during health care consultations [38,39]. Therefore, an assessment of patient activation focused on interactions within consultations could be instructive to health care professionals.

The CICS classifies interactions into themes or interaction types (eg, *action planning*) and assigns a rating to each interaction type based on the level of patient activation. Higher scores denote greater patient activation. Ratings on the CICS have been shown to be associated with working alliance, therapist competence, multiple physical and mental health outcomes, and important clinical changes within therapy among patients with multimorbidity receiving psychological therapy over videoconferencing [32,40,41]. The CICS could address some of the key gaps in AI use for psychological therapy, particularly among patients with multimorbidity and in applications of remote psychological therapy. It may, therefore, offer a basis for an explainable, automated psychological therapy rating tool.

### Aims

This study’s aims were as follows:

1. Involve end users and stakeholders in applying participatory elements of an explainable AI methodology to coproduce an initial, automated version of the CICS (autoCICS).
2. Assess the performance of the autoCICS ratings compared with human rating reliability.
3. Identify key language features associated with high and low patient activation as well as different interaction types.

Overall, a participatory methodology, which helps to build trust among stakeholders, was applied to the responsible design and development of an autonomous psychological therapy rating system.

## Methods

### Data Source

Source data included 128 hours of audio data from remotely delivered cognitive behavioral therapy (rCBT) from 53 participants in a randomized controlled trial of rCBT versus usual care for people with severe health anxiety using urgent care at a high rate [42]. Participants were randomly allocated to rCBT plus usual care (n=79) or usual care alone (n=77). There were 78 participants randomized to rCBT, and 1 participant was randomized to usual care but offered rCBT in error. Their data are included in the analysis. Therefore, the total sample is 79. Participants randomized to rCBT were offered up to 15 sessions of rCBT delivered via videoconferencing software (54/79, 68%) or the telephone (14/79, 18%; the remaining participants—11/79, 14%—did not attend any sessions). Most of the participants were not seeking psychological therapy when recruited (69/79, 87%), and most reported multimorbidity (75/79, 95%).

The randomized controlled trial recruited 156 participants from UK primary and secondary health care settings. Participants were adults (aged ≥18 years) who had received ≥2 unscheduled or urgent consultations with any health care provider in the previous 12 months and were identified as highly anxious about their health. Participants were excluded if they were experiencing an acute medical condition requiring ongoing assessment, but those with comorbid common mental health problems or chronic physical conditions such as depression or chronic pain were intentionally included.

Of the 79 possible participants, 53 (67%) were included, having (1) attended ≥1 rCBT sessions and (2) consented to treatment sessions being recorded and extracts anonymously reported. The structured clinical interview for the Diagnostic and Statistical Manual of Mental Disorders, Fourth Edition [43], was completed with participants at baseline, assessing for criteria of mental disorders. Long-term physical health conditions were also recorded from baseline patient interviews.

Four psychological therapists delivered rCBT using an established treatment protocol [42]. Of the 4 therapists, 2 were women, and 2 were men; 2 had doctoral-level clinical psychology training, and 2 had master’s-level psychological therapy training.

Of the 128 included sessions, 98 (76.5%) were first and second sessions, and 30 (23.4%) were identified as sessions of potential clinical importance: occurring directly before a sudden sustained improvement, sudden deterioration, or dropout or were the center session in a series where little or no outcome change occurred. The group of 98 sessions (total 42,064 turns of speech) was used to develop and train the initial model, and the other 30 sessions (total 9,239 turns of speech) were used as a holdout sample to test the model once developed. This split fitted with the separation of early sessions and clinically relevant later sessions available. It also approximated to the established 80:20 percentage split for training and test data sets.

### Ethics Approval

Ethics approval was obtained from the National Research Ethics Service, London-Riverside Committee (14/LO/1102).

### CICS Categories

The CICS categorizes each in-session turn of speech and rates the level of patient activation. A turn of speech is defined as the words spoken by one party until the other party speaks; when the other speaker begins speaking, the first speaker’s turn of speech is deemed to have ended. First, a topic is assigned for the turn of speech from ≥1 of the CICS themes using observable criteria (Textbox 1).

Once an interaction theme is allocated, the level of patient activation present in this interaction is rated. Scores range from +2 for interactions showing observable, high levels of patient activation and engagement to −2 for interactions showing observable indications of low patient activation and disengagement. The CICS rating level allocated is linked to established levels of patient activation (Table 1 presents overall level descriptors for CICS themes and comparator patient activation levels; Table 2 presents an example of level descriptors for the *evaluations of self or therapy* theme). The 2 higher levels of patient activation (3 and 4, equivalent to CICS +1 and +2) are linked with positive health outcomes, and the 2 lower levels (1 and 2, equivalent to CICS −2 and −1) are associated with poorer health outcomes across a range of domains [44]. The CICS coders were trained using a published manual [45].

CICS ratings are defined on the basis of a therapist-patient interaction combined. This aims to address the key issue of responsiveness in psychological therapy. Therapist responsiveness is defined as behavior that is influenced by emerging context, such as a therapist changing their verbal response in line with changes in patient presentation [46]. This kind of responsiveness is an important contributor to therapists’ effectiveness [47]. Accounting for this type of responsiveness aims to give therapists feedback on their behavior within specific patient contexts; for example, previous machine learning studies of text-based psychological therapy have identified therapeutic praise (eg, “Well done”) from therapists as predictive of better outcomes [11]. However, these therapist utterances must occur in the context of specific patient interactions, which is not accounted for when only the therapist response is considered.

All CICS themes have achieved good-to-excellent interrater reliability (intraclass correlation coefficients=0.60-0.80), and most achieved convergent validity with cognitive behavioral therapy competence and working alliance (*r_s_*s=0.72-0.91). The *problem or context description* interaction theme (rated present or absent) has shown moderate-to-substantial interrater reliability (κ=0.54-0.61) and negative associations with working alliance and therapist competence (*r_s_*=−0.71 and −0.47) [32].

**Description of consultation interactions coding scheme themes.**

**Interaction theme and description**

- Action planning and idea generation: discussion of specific plans or potential plans for activities outside the session
- Evaluations of self or therapy: offering a personal assessment of therapy or of one of the parties in therapy
- Information discussion: giving, receiving, or requesting specific information
- Noticing change or otherwise: where changes are reported that relate to therapeutic work, or a lack of change is described despite efforts to bring it about
- Other: where interactions were not related to therapy; most commonly, these interactions involved resolving technical issues associated with videoconferencing
- Problem analysis and understanding: an analysis or understanding of a problem is given or received
- Problem or context description: description of problems or contexts surrounding problems
- Structuring and task focus: where verbal efforts to structure, plan, or progress the session are offered or sought; conversely, where sessions deviate from any relevant topic without intervention from either party

**Table 1:** Consultation interactions coding scheme (CICS) scores and equivalent, mapped patient activation levels (adapted from the study by Deeny et al [20]).

CICS level	CICS-level descriptor	Mapped PAM^a^ level	PAM-level descriptor and percentage of patients at each level^b^
+2	A high level of patient activation and focus is observable; an interaction usually led by the patient. This would include patient-initiated therapeutic activity not cued or primed by the previous therapist interaction	4	Level descriptor: “I’m my own advocate.” Patients who are confident in developing and adopting behaviors and practices to manage their health, such as care planning or self-monitoring. Such individuals may be connecting with supportive others (13% of respondents)
+1	Significant patient activation is observable but with less leadership. Typically, this would be a therapeutically active interaction, led or guided by the therapist, which the patient endorses and develops with their contributions	3	Level descriptor: “I’m part of my health care team.” Patients who seem to be taking action, for example, setting goals for their health (such as adhering to a medically advised diet) or collaborating in development of a care plan with health care providers, but may still lack the confidence and skill to maintain these (46% of respondents)
0 or neutral	These are interactions where few or no observable positive or negative interaction features are apparent with regard to patient activation. These interactions are deemed to be neutral—neither beneficial nor detrimental to the outcome. The same code is applied if a theme is absent. This includes interactions where therapists make suggestions or comments with little or no observable sense of how the patient receives them	N/A^c^	N/A
−1	Hypothesized to be therapeutically unhelpful interactions in a minor way. This includes interactions that show the start of unaddressed disagreements or reluctance to engage with therapeutic activities. Low levels of patient activation and involvement are observed	2	Level descriptor: “I could be doing more.” Patients who may manage some low-level aspects of their health but struggle in many aspects of their care, such as engaging with care planning (19% of respondents)
−2	Hypothesized to be interactions that would be contradictory to most therapeutic guidance. This would include argumentative or obstructive interactions where the patient and potentially the therapist appear disengaged, unfocused, and oppositional to therapeutic activity	1	Level descriptor: “My clinician is in charge of my health.” Patients tend to feel overwhelmed by managing their own health and may not feel able to take an active role in their own care. They may not understand what they can do to manage their health better and may not see the link between healthy behaviors and good management of their condition (22% of respondents)

^a^PAM: patient activation measure.

^b^Data taken from a UK sample of 9348 primary care patients [20].

^c^N/A: not applicable.

**Table 2:** Level descriptors and exemplar quotes for the evaluations of self or therapy consultation interactions coding scheme theme.

Level	Level descriptor^a^	Exemplar quote
+2	Patient-initiated statements of *self-efficacy,* patient *acknowledgment or pride* at therapeutic achievement, or *positive evaluations* of therapy or the therapist that are initiated by the patient	Patient: “Like I had a panic attack on Friday so randomly...and I was so good, like I dealt with it so well...I was so good at sort of, like, joking around with myself and I was like yeah, just stay here, just like breathe, like, and I remember thinking, like, I know that, like, no one, these people sitting next to me, literally have no idea because part of me was just like right just carry on because it’s going to pass, it’s going to pass.” Therapist: “Yeah.” [P01078]
+1	Therapist-initiated positive evaluation; as in the previous row, patient agrees with *development and summary or* *corrections*	Therapist: “It sounds like you did exactly the right thing...how you addressed your worry; you know reflecting on it and actually, you know, taking action...rather than just sitting ruminating and going deeper into worry. Sounds like you did the right thing.” Patient: “Yeah. I think reflecting is the best thing I ever did because I was so scared, I was so worried about the outcome...but when I looked at it, it’s not my responsibility.” [P01108]
0	Therapist-initiated positive evaluation; patient acknowledges with *no development* or very low–level acknowledgment by the patient	Therapist: “You handled those thoughts well by, you know, not letting them become more catastrophizing by recognizing for what they were and managing to handle them pretty well.” Patient: “Mmm.” [P01096]
−1	Therapist’s positive evaluations, as in the previous row, are *undermined to some degree* by the patient or *somewhat negatively focused* self-evaluations or statements about therapy or therapist	Therapist: “Yeah, that’s huge. How do you feel about yourself, given that you’ve done all this stuff this week?” Patient: “Well, I’m really pleased with this week, but I’m still cross about the things that I didn’t do, as opposed to being pleased about the things that I did do.” [P03014]
−2	*Self-denigrating or self-critical* statements or a *self-critical focus* on therapeutic tasks that have not been completed to the exclusion of those that have been completed by the patient	Patient: “I wouldn’t say that I have that much control over my way of dealing with things.” Therapist: “Really?” Patient: “Yes.” [P01007]

^a^Italics add emphasis to the key component of the level descriptor.

### Focusing on Problem or Context Description

The most reliable finding from predictive modeling with the CICS so far is that the greater the proportion of sessions taken up with *problem or context description* interactions, the poorer the outcome. In this way, *problem or context* *description* interactions were predictive of poorer generalized anxiety, health anxiety, depression, quality of life, and general health across a 12-month follow-up [41]; they also negatively predicted well-being rated across therapy sessions and significantly reduced in frequency directly before sudden sustained outcome improvements [40]. Despite being associated with poorer outcomes, *problem or context description* interactions are conceptualized as neutral, not negative, interactions—describing problems is a necessary and normal part of psychological therapy; however, excessive focus on problem description alone may crowd out space for other types of interactions, particularly those where higher patient activation is indicated and greater active engagement may be stimulated. Therefore, *problem or context* *description* interactions are scored present or absent as opposed to higher or lower patient activation as in the case of other interaction themes, with the aggregate score being the percentage of the session rated for the theme.

Given the central importance of *problem or context description* interactions to the prognostic validity of the CICS, we first focused autoCICS classification modeling on identifying *problem or context description* interactions versus other interactions. Second, given the importance of higher patient activation across the other CICS interaction themes, autoCICS classification modeling also focused on identifying interactions categorized as higher versus lower levels of patient activation.

### Data Preprocessing

Each session was transcribed verbatim, with any identifying information removed during transcription, and transcripts were then checked for anonymity by the raters. Each transcribed turn of speech was coded in NVivo software (version 12.0; QSR International) by three trained raters using the CICS (SM, CM, and NM). A third pass was carried out in preprocessing to assign a master code to each turn of speech accounting for the previous raters’ decisions. Overlapping codes were also removed in the master code because they would not be processed effectively when generating classification models in the autoCICS approach. The two possible positive ratings on the CICS (+1 and +2) were collapsed into a single positive category (1), and the possible neutral and negative ratings (0, −1, and −2) were collapsed into a single negative category (0), sacrificing some granularity in the data to increase data subgroup sizes used to train the predictive models. General demographic features were added as predictor variables alongside language features, including participant age and sex, alongside therapist sex. Features were also added to represent the natural grouping of transcribed speech: speech from the same patient, as well as interactions occurring at the beginning, middle, or end of a session (dividing the total turns of speech into three). Minimal demographic features were used with the aim of both addressing common end-user concerns about data security, particularly with such sensitive data being used, and minimizing potential to propagate biases in AI systems [48,49]. Language features were excluded where all values were zero. For models classifying interaction themes, original CICS codes were converted to *problem or context description* interactions versus other interaction themes combined.

### Coproduced Linguistic Feature Extraction

The autoCICS development team was deliberately assembled to ensure that it comprised key research and clinical stakeholders with regard to the characteristics of an automated psychological therapy rating tool. The team comprised 2 psychological therapists and a psychological therapy assistant (SM, NM, and CM, respectively), who offered clinical expertise; 3 service-user researchers (MR, FH, and DW), who offered patient-related knowledge and experience; an applied linguist (DH), who contributed expertise on linguistic functions and patterns; an AI ethics researcher (EPV); and an explainable AI researcher (JC), who added an understanding of how participatory methodology could be meaningfully translated into NLP features. The team members were separately surveyed about what language markers in patient-therapist interactions they thought might be indicative of greater patient activation—that is, active engagement, involvement, and ownership of the therapeutic process. The team members were also asked what language markers they felt might indicate a patient’s disengagement and withdrawal from therapeutic processes. The features identified were then collaboratively translated into NLP features by three other team members: an NLP researcher (YL) and two digital research experts (TJ and GF). Table 3 presents examples of the language features suggested by different disciplinary groups within the team (refer to Multimedia Appendix 1 for the final language features used in validation with nonsignificant features removed). This process aimed to generate understandable language features from different relevant perspectives for the future product’s end users. This methodology aimed to enhance transparency and involve domain experts in selecting input features rather than unsupervised learning from the data, which would likely be less interpretable. Language features were extracted using the Python Natural Language Toolkit (NLTK Project) and the Python library, TextBlob.

**Table 3:** Examples of suggested language features deemed indicative of greater patient activation.

Suggestion source and language feature		Related study
**Service users**		
	Less profanity (swear words and curses)	Coppersmith et al [50]
	Fewer absolutes (always, never, and everything)	Al Mosaiwi and Johnstone [51]
	Fewer maximizers (worst and most)	Strohm and Klinger [52]
**Psychological therapists**		
	Positive sentiment (happy, glad, and good)	Calvo et al [53]
	Intensity of positive sentiment (polarity and frequency)	Calvo et al [53]
	Lower ratio of illness: wellness terminology	Arseniev et al [54]
**Applied linguist**		
	Fewer deontics (eg, must, should, and ought)	Van der Zanden [55]
	Fewer qualifier words (eg, but and though)	Jeong [56]
	Ratio of plural: singular first-person pronouns	Rude et al [57]
**Explainable artificial intelligence researcher**		
	Longer sentences (number of words)	Hirschberg et al [58]
	Longer words (number of characters)	Pestian et al [59]
	Lower Flesch-Kincaid readability score (more complex sentences)	Pestian et al [59]

### Machine Learning

A bagged trees algorithm was used to classify patient activation level, that is, differentiating between interactions rated positively (+1 or +2) and those rated negatively or neutral (−1, −2, or 0). The model used a constant weight of 3 for misclassified instances at level 1 to penalize misclassifications in the less frequent class. The constant of 3 was reached through algorithm optimization during training. A k-nearest neighbor algorithm was used to classify interaction types; specifically, differentiating between *problem and context description* interactions and other interaction types, given the prognostic importance of these interactions. Both models were developed using MATLAB (version 2021a; MathWorks, Inc). The standard implementation from MATLAB uses hyperparameter tuning intrinsically. Exploratory modeling also evaluated the classification of other, less frequent interaction types rated on the CICS (eg, *evaluations of self or therapy*). The synthetic minority oversampling technique [60] was initially applied to augment the data, but it did not significantly improve the results; therefore, it was removed, particularly given that highly unbalanced data set and potential clinical use.

## Results

### Sample Characteristics

The included participants were predominantly White British (40/53, 75%), and three-quarters (40/53, 75%) were female. All participants had been assessed as experiencing severe health anxiety using the short health anxiety inventory, but all participants reported additional comorbidities. On average, participants met criteria for 7 (SD 3.7) mental disorders from the structured clinical interview for the Diagnostic and Statistical Manual of Mental Disorders, Fourth Edition, assessment, most commonly generalized anxiety disorder. Participants also reported a mean 1 (SD 1.15) additional chronic physical health condition, most commonly chronic pain (refer to Table 4 for participant demographics and clinical characteristics).

**Table 4:** Demographics and clinical characteristics of participants (N=53).

Variable							Values
**Demographics**							
	Sex, female, n (%)						40 (75)
	Age (years), mean (SD)						36 (15)
	**Ethnicity, n (%)**						
			White British			40 (75)	
			Other			13 (24)	
	Unemployed, n (%)						6 (11)
**Clinical characteristics**							
	**SCID^a^ diagnoses, mean (SD; range)**					7 (3.7; 0-16)	
				Generalized anxiety disorder, n (%)		35 (66)	
				Hypochondriasis, n (%)		34 (64)	
				Somatoform disorders, n (%)		33 (62)	
				Current depressive episode, n (%)		32 (60)	
				Panic disorder, n (%)		32 (60)	
	**Long-term physical health problems, mean (SD; range)**					1 (1.15; 0-6)	
					Chronic pain, n (%)	13 (25)	
					Chronic fatigue, n (%)	5 (9)	
					Functional neurological disorders, n (%)	5 (9)	
					Irritable bowel syndrome, n (%)	4 (8)	
					Arthritis, n (%)	4 (8)	
					Diabetes, n (%)	4 (8)	

^a^SCID: structured clinical interview for the Diagnostic and Statistical Manual of Mental Disorders, Fourth Edition.

### Data Characteristics

*Problem or context description* interactions were the most commonly coded CICS theme, accounting for 54.6% (22,967/42,064) of the interactions in the training data set and 46.8% (4324/9239) of the interactions in the test data set. Conversely, interactions involving patients’ *evaluations of self or therapy* were the least coded interaction type, accounting for 2.4% (1010/42,064) and 3% (277/9239) of the training data set and test data set, respectively.

### Interaction Classification

Given that the data set was imbalanced, *F*-scores are reported alongside accuracy scores because they are less sensitive to class imbalance. For the model based on a k-nearest neighbor algorithm used to identify CICS-rated interaction themes (correctly identifying *problem or context description* interactions versus other interactions), an overall accuracy of 73% (precision=82%, recall=80%, and *F*-score=73%) was observed in the test data set. The model used to classify the CICS-rated patient activation level (positive versus negative or neutral) obtained an 81% accuracy (precision=87%, recall=75%, and *F*-score=87%) in the test data set.

Exploratory models aiming to classify less frequent interaction themes (*action planning and idea generation*, *evaluations of self or therapy*, *information discussion*, *noticing change or otherwise*, *problem analysis or understanding*, and *structuring and task focus*) obtained lower-than-average *F*-scores of 20% because of very high class imbalance.

## Discussion

### Principal Findings

This study indicates that collaboratively and transparently developed AI can be used to discriminate between high and low patient activation from turns of speech in psychological therapy sessions. The language features used also discriminated between *problem or context description interactions* and other interaction types. However, the model could not discriminate among other interaction types on the CICS (eg, *action planning* versus *problem analysis or understanding*). The codevelopment approach applied may help to improve trust in the decisions made by an autoCICS psychological therapy rating tool among end users, including patients, psychological therapists, and service managers [31]. The model was also enhanced by including key stakeholders in the selection of language features that formed the basis of the prediction models, rather than using an exclusively data-driven approach likely to end in more opaque and potentially spurious processes that have reduced trust in AI generally [48]. The involvement of stakeholders in this way also helps to develop a fit-for-purpose system within health care when AI applications often lack adequate end-user involvement [61]. Overall, the findings suggest that reasonable predictive accuracy was achieved with the participatory methodology applied (involving key stakeholders in the AI model development).

### Comparison With Prior Work

By including participatory approaches to enhance trust and interpretability, this study builds on existing research where AI has been used to automate psychological therapy rating tools [10,62]. Similar levels of agreement with human rating reliability were achieved in this study compared with previous attempts to automate psychological therapy turn-by-turn ratings [9,63]. This suggests that the simplifications made to the modeling for greater interpretability have not been excessively detrimental to model performance. An automated assessment that takes account of both therapist and patient utterances in this study may also help build a clearer understanding of language features associated with therapeutic responsiveness in future [47]. This is particularly relevant because many current machine learning models focus on either therapist or patient utterances alone [9,11]. Whereas most previous supervised learning models have focused on in-session behaviors related to a specific therapeutic model (eg, motivational interviewing [10]), the autoCICS in this study assesses patient activation—a construct that may have relevance across psychological therapy models and treatments in other domains [64]. Furthermore, this study expands the range of patients included in this type of modeling with a patient sample experiencing multimorbidity at baseline. Given the importance of health care professionals’ interaction style and responsiveness to enhance patient activation during consultations with people experiencing multimorbidity, an automated interaction assessment has potential for broad application in improving care [21]. By including the now often used modality of remote psychological therapy, this study also expands the range of psychological therapy delivery modalities where NLP has been applied.

### Limitations

This study used a relatively small sample size for machine learning studies. This means that the breadth of interaction types and language features used may be restricted, making the results less generalizable. However, the sample size is typical compared with previous studies of NLP in psychological therapy [65]. The smaller sample size also limited use of more complex modeling methods that could have improved classification precision and sensitivity, especially when considering more levels of granularity with regard to the interaction types and patient activation levels. Relatedly, a limited number of therapists were included in the data set; a more representative sample of therapists may have helped identify and define important differences among therapists who could be included in models to improve accuracy. A larger number of therapists could also help to discriminate among different clustered therapist phenotypes, where different interaction styles could be attributed to specific therapist groups.

In exploratory modeling, the classifier accuracy in less frequent classes of interaction was much lower. This suggests that either there was insufficient data to train the model, or the language features applied in the models did not discriminate among these interaction themes very well. The result is that the current classifier could not offer refined, granular feedback to practitioners on more detailed aspects of their session contents. Another possible explanation for the classifier’s poor performance in discriminating among different interaction types (eg, structuring interactions versus information giving) is that the same language features were used to classify both patient activation level and interaction type. Different language features may have given clearer differentiation on interaction types.

Although the CICS-labeled data used to train the model in this study aimed to address therapist responsiveness by combined ratings of therapist and patient data, this prevents an understanding of individual contributions to patient activation from either therapist or patient; for example, where a patient’s interaction indicates movement toward greater engagement, but the therapist’s response undermines this. The current classification process would struggle to identify these occasions, which could be important for therapist feedback.

Although this study indicates that the autoCICS achieved good discriminative validity, it is unclear whether this would be sufficiently accurate for reliable use in clinical settings. Furthermore, the practical, clinical value of the classifier would need to be evaluated in practice before significance could be assessed. Therefore, further model validation is required, and the feasibility and acceptability of the tool in clinical practice should be assessed, given the catalog of implementation failures for AI tools in health care more broadly [24].

### Future Research

The automated ratings presented in this paper require external validation to clarify whether interactions rated as high in patient activation associate with assessments of patient activation used in clinical practice, such as the PAM, conducted at the same time point. The clinical utility of the automated assessment cannot be assured until such validation has been carried out.

Larger-scale validation could use a varied, more representative patient and therapist sample to help improve the generalizability of the model and address potential biases in model decisions. Future research may also benefit from use of routine care data sets (in contrast to research trial data, as in this study). This may give a closer representation of therapeutic processes experienced in real-world therapy and, therefore, increase wider applicability. Validation across different psychological therapy models and presenting problems would also help to establish transferable aspects of the model’s utility. Future research should also clarify the prognostic value of the autoCICS not only to establish whether sufficient reliability has been achieved to retain the CICS predictive validity but also to assess whether predictive validity can be improved using a codevelopment approach.

This study, alongside most previous research, has focused on lexical elements of psychological therapy content (transcribed words), but it does not address the nonlexical, phonological features of talk (such as intonation and prosody) that can be an important predictor of health [66]. Therefore, future research should address the integration of lexical and phonological analyses of psychological therapy content for more accurate representations of in-session events. Finally, future research should identify means of building and maintaining codevelopment, interpretability, and transparency within more complex AI analyses of psychological therapy content. Collaboratively developed models may not identify the same features as either expert-designed models or unsupervised learning models, but they may be more trustworthy and fit for purpose for end users [29]. In future, contrasting results from participatory approaches, such as the one used in this study, with more *black box* approaches to developing an automated classifier would give an informed view on the trade-off between model accuracy and algorithmic trust. This will be particularly important if greater accuracy is to be achieved in classifying more detailed interaction types, which could not be achieved with the current methodology. Importantly, the participatory methods used do not preclude the use of more complex algorithms to develop models in future research.

### Clinical Implications

This study presents the initial development of an automated assessment of patient activation that can be rated turn by turn routinely in psychological therapy. Alongside other advances, this methodology may help enhance deliberate practice techniques in psychological therapy. Deliberate practice aims to identify therapeutic microskills requiring improvement and then improve these skills through corrective practice [16]. In conjunction with a further developed autoCICS, alongside associated training and supervision, therapists could learn to recognize problematic patterns more easily and practice different responses.

### Conclusions

A participatory methodology was applied to develop a novel approach for the assessment of in-session patient activation during psychological therapy. This approach can support the responsible design and development of autonomous and intelligent systems in psychological therapy by building trust among stakeholders from initial development.

Language features identified by a multiperspective stakeholder collaboration can be used to discriminate between high and low patient activation in psychological therapy session contents but were limited in their ability to discriminate among different psychological therapy interaction types. However, larger-scale replication is required before clinical utility can be assessed.

## Appendix

Multimedia Appendix 1 Final language features used in modeling.

## Abbreviations

AI: artificial intelligence
autoCICS: automated consultation interactions coding scheme
CICS: consultation interactions coding scheme
NLP: natural language processing
PAM: patient activation measure
rCBT: remotely delivered cognitive behavioral therapy
