# Supplementary material for: Developing an Automated Assessment of In-session Patient Activation for Psychological Therapy: Codevelopment Approach
Source: JMIR Med Inform. 2022 Nov 8;10(11):e38168. doi: 10.2196/38168 (PMC9682451; doi:10.2196/38168)
Supplement: Multimedia Appendix 1 [file medinform_v10i11e38168_app1.docx]

Multimedia appendix 1

Supplementary Table 1. Final language features used in modelling

| **Language Feature** | **Description** |
| --- | --- |
| Sentiment | Positive, negative or neutral sentiment expressed |
| Polarity | The strength of positive or negative sentiment |
| Sentence word length | Number of words in a sentence |
| Sentence character length | Number of characters in a sentence |
| Flesch-Kincaid readability score | Complexity of the language used |
| Number first person singular pronoun | Use of I, me, my, mine, or myself |
| Number first pl person pronoun | Use of we, us, our, or ourselves |
| Number second person pronoun | Use of you, your, yours, yourself, or yourselves |
| Number third person pronoun | Use of he, she, it, they |
| Number qualifier words | Number of words used to indicate uncertainty (e.g. probably, possibly, sometimes) |
| Number exclusivizers | Use of words that minimise or psychologically distance (e.g. just, only) |
| Number illness words | Use of words that indicate illness |
| Number wellness words | Use of words that indicate wellness |
| Number detrimental outcome | Language suggesting an unwanted outcome (e.g. it went badly) |
| Number agreement words | Use of words that suggest agreement with the other party (yes, that's right) |
| Position | Whether the speech occurred in the first, second or final third of the session |
| Patient gender | Gender of patient |
| Patient age | Age of patient |
| Therapist gender | Gender of therapist |

Note: Excludes language features included in training, but removed in validation due to weak predictive validity
